# Prognostic implication of late gadolinium enhancement on cardiac MRI in light chain (AL) amyloidosis on long term follow up

**DOI:** 10.1186/1756-6649-9-5

**Published:** 2009-05-05

**Authors:** Raymond Q Migrino, Richard Christenson, Aniko Szabo, Megan Bright, Seth Truran, Parameswaran Hari

**Affiliations:** 1 Cardiovascular Medicine Division, Medical College of Wisconsin, 9200 W Wisconsin Avenue, Milwaukee WI 53226, USA; 2 Radiology Department, Medical College of Wisconsin, 9200 W Wisconsin Avenue, Milwaukee WI 53226, USA; 3 Biostatistics Division, Medical College of Wisconsin, 9200 W Wisconsin Avenue, Milwaukee WI 53226, USA; 4 Hematology-Oncology Division, Medical College of Wisconsin, 9200 W Wisconsin Avenue, Milwaukee WI 53226, USA

## Abstract

**Background:**

Light chain amyloidosis (AL) is a rare plasma cell dyscrasia associated with poor survival especially in the setting of heart failure. Late gadolinium enhancement (LGE) on cardiac MRI was recently found to correlate with myocardial amyloid deposition but the prognostic role is not established. The aim is to determine the prognostic significance of LGE in AL by comparing long term survival of AL patients with and without LGE.

**Methods:**

Twenty nine consecutive patients (14 females; 62 ± 11 years) with biopsy-proven AL undergoing cardiac MRI with gadolinium as part of AL workup were included. Survival was prospectively followed 29 months (median) following MRI and compared between those with and without LGE by Kaplan-Meier and log-rank analyses.

**Results:**

LGE was positive in 23 subjects (79%) and negative in 6 (21%). Left ventricular ejection fraction was 66 ± 17% in LGE-positive and 69 ± 12% in LGE-negative patients (p = 0.8). Overall 1-year mortality was 36%. On follow-up, 14/23 LGE-positive and none of LGE-negative patients died (log rank p = 0.0061). Presenting New York Heart Association heart failure class was also associated with poor survival (p = 0.0059). Survival between two LGE groups stratified by heart failure class still showed a significant difference by a stratified log-rank test (p = 0.04).

**Conclusion:**

Late gadolinium enhancement is common and is associated with poor long-term survival in light chain amyloidosis, even after adjustment for heart failure class presentation. The prognostic significance of late gadolinium enhancement in this disease may be useful in patient risk-stratification.

## Background

Light chain or primary amyloidosis (AL) is a plasma cell dyscrasia with production and abnormal deposition of insoluble fibrillar proteins derived from immunoglobulin light chains [1,2]. It is a rare disease but can be fatal. Amyloid protein deposition in various organs such as the heart, kidneys, gut and nervous system cause multi-organ dysfunction. Cardiac involvement was reported in 50% of cases [3] and is associated with the worst prognosis; median survival is about 4 months in the setting of advanced heart failure [4,5]. Amyloid deposition in the heart is confirmed by invasive endomyocardial biopsy. Noninvasive imaging demonstrate that some AL patients have diffuse subendocardial late gadolinium enhancement (LGE) on cardiac magnetic resonance imaging (MRI) [6]. Late gadolinium enhancement was found to be highly correlated to histologic evidence of myocardial amyloid deposit [7] pointing to its diagnostic potential in assessing cardiac involvement. Recently, gadolinium kinetics assessed by intramyocardial (subepicardium and subendocardium) difference in T1 was found to predict mortality in AL patients; the presence of LGE showed a trend, but not significant, of increased mortality [8]. To further define the prognostic role of LGE in light chain amyloidosis, the aim of our study is to compare the long-term survival of AL subjects with and without LGE.

## Methods

### Patient population and clinical data

Among 36 consecutive patients with biopsy-proven diagnoses of light chain amyloidosis seen by our Oncology or Cardiology Division from April 2005–November 2008, 29 underwent cardiac MRI as part of routine workup and were included in this study. Seven patients did not undergo cardiac MRI because of low glomerular filtration rates unsuitable for gadolinium administration and were not included in this study. The study was approved by the local Institutional Review Board (IRB). Twenty five patients signed informed consent as part of a longitudinal study of light chain amyloidosis and were prospectively followed. Four patients undergoing workup expired before recruitment for the study and waiver of consent was authorized by the IRB for data collection. Their data were included to ensure 100% capture of consecutively evaluated AL subjects in our institution. Clinical data was obtained that included presenting hemodynamic, laboratory profile and New York Heart Association heart failure functional class (I–IV). The latter was adjudicated by a cardiologist based on presenting symptoms and signs according to established clinical standards [9]. Low voltage on electrocardiogram was defined as ≤ 5 mV in all limb leads [10]. For analysis, troponin I was dichotomized using cutoff values of upper limits of normal in our institution (0.1 ng/mL) and alkaline phosphatase was dichotomized using 1.5 times upper limit of normal for our laboratory following previously published cutoff thresholds [11]

### Cardiac MRI

A General Electric 1.5 Tesla CV scanner was used with 8-channel cardiac coil. Cardiac gated cine segmented steady state free precession pulse sequence was performed on short axis view orthogonal to the long axis of the left ventricle to assess left ventricular function by manual tracing of endocardial borders using ReportCard software (General Electric, Waukesha WI). The imaging parameters used were: field of view 34–40 cm, 160 × 256 matrix, 7–8 mm slice thickness, 2–3 mm gap, flip angle 45°, retrospective gating. For late gadolinium enhancement imaging, 0.1 mmol/kg of gadolinium (gadodiamide, GE Healthcare or gadobenate dimeglumine, Bracco Diagnostics) was injected and imaging started after ~5 minute delay in short axis and multiple long axis views. Cardiac gated segmented inversion-recovery prepared gradient echo pulse sequence was used with field of view 38–42 cm, matrix of 256 × 192–256, slice thickness of 7–8 mm, interslice gap of 2–3 mm, inversion time of 175–300 ms adjusted to null normal myocardial signal, number of excitations of 1–2 and 2 R-R intervals. The optimal inversion time that nulls normal myocardium was determined by acquiring multiple images of the same midventricular view using different inversion times. If a significant portion of the myocardium goes through the null point earlier than the left ventricular cavity blood pool this area was considered to represent myocardial disease and subsequently the inversion time was then adjusted to null the remaining myocardial areas (usually in subepicardial region), an approach that has been previously validated with histologic gold standard [7]. Two expert readers of cardiac MRI independently adjudicated the presence or absence of LGE with agreement of the findings in all cases.

### Data and Statistical Analyses

Data are expressed as mean ± standard deviation. Continuous variables were compared between groups using Student's t-test (for normal distribution) or Mann-Whitney rank sum test (for non-normal distribution) using SigmaStat 3.5 software (Systat Software, Inc., Point Richmond CA). Categorical variables were compared using Fisher's exact test. Stratified estimates of survival probabilities were computed using Kaplan-Meier estimators [12] with log-rank test used to test for group differences in survival curves. To assess the interaction between heart failure class and LGE, an exact permutation test based on stratified log-rank test was used to compare the two LGE groups stratified by heart failure class (MedCalc 9.6.4.0, Mariakerke, Belgium, SAS 9.1.3, SAS Institute Cary NC). Cox multivariable analysis was not performed because of non-estimable parameters (specifically, the absence of mortality in the late gadolinium enhancement negative group does not allow a realistic estimation of the hazard ratio in Cox regression models) and otherwise unstable parameter estimates with our small sample size. A p-value less than 0.05 was used to denote statistical significance.

## Results

### Patient population and clinical characteristics

**Figure 1:**
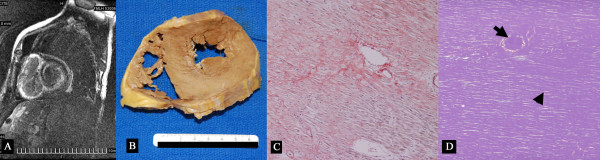
**Light chain amyloidosis case**. A 76 year old with cardiac involvement who had electromechanical dissociation and heart failure and expired despite melphalan chemotherapy. A. Cardiac MRI following gadolinium injection demonstrates subendocardial delayed enhancement in the left ventricle and transmural delayed enhancement in the right ventricle. B. Gross specimen showing thick left and right ventricles. C and D. Congo red stain without (C) and with polarized light (D) demonstrates diffuse interstitial infiltration (arrowhead) and perivascular infiltration (arrow) of amyloid substance.

There were 14 females with mean age of 62 ± 11 years. Biopsy was positive for amyloid in the heart (N = 4, Figure 1), kidneys (N = 14), bone marrow (N = 8), fat pad (N = 5), liver and hip bone (N = 2 each), tongue, gastrointestinal tract and axillary mass (N = 1 each) and all subjects had abnormal elevation of kappa or lambda light chains in serum, urine or bone marrow. Thirteen (45%) had NYHA heart failure class I with 10 (34%), 1 (3%) and 5 (17%) in NYHA heart failure class II, III and IV respectively. The baseline clinical characteristics of the subjects are described in Table 1. There was no significant difference in presenting vital signs or laboratory indices between LGE-positive and LGE-negative patients. There was a trend which is not significant towards higher proportion of low voltage electrocardiogram in LGE-positive patients. 91% of LGE-positive and 100% LGE-negative subjects underwent chemotherapy with or without autologous stem cell transplantation (Fisher's exact p = 1.0). Two (out of 23) LGE-positive patients did not have chemotherapy: 1 refused chemotherapy and the other had unstable heart failure.

**Table 1:** Clinical characteristics of light chain amyloidosis patients.

	**LGE+ (N = 23)**	**LGE- (N = 6)**	**p-value**
Age (years)	63 ± 12	58 ± 9	0.35
Heart rate (beats per minute)	82 ± 21	71 ± 28	0.26
Systolic blood pressure (mm Hg)	117 ± 18	141 ± 39	0.2
Chemotherapy/Stem cell transplant (N/%)	21 (91)	6 (100)	1.0
NYHA Heart Failure Class (N/%)			0.19
I	8 (35)	5 (83)	
II	9 (39)	1 (17)	
III	1 (4)	0	
IV	5 (22)	0	
**Laboratory**			
Low voltage on ECG (N/%)	15 (65)	1 (17)	0.06
Alkaline phosphatase (U/L)	141 ± 151	75 ± 23	0.13
Troponin I (μg/L)	0.51 ± 0.6	0.23 ± 0.3	0.33
**MRI**			
Left ventricular ejection fraction (%)	66 ± 17	69 ± 12	0.8
Anteroseptal thickness (mm)	1.5 ± 0.4	1.12 ± 0.4	0.05
Inferolateral thickness (mm)	1.3 ± 0.4	0.9 ± 0.2	**0.0003**
Left ventricular mass index (g/m^2^)	81 ± 30	61 ± 10	**0.01**

### Cardiac MRI

**Figure 2:**
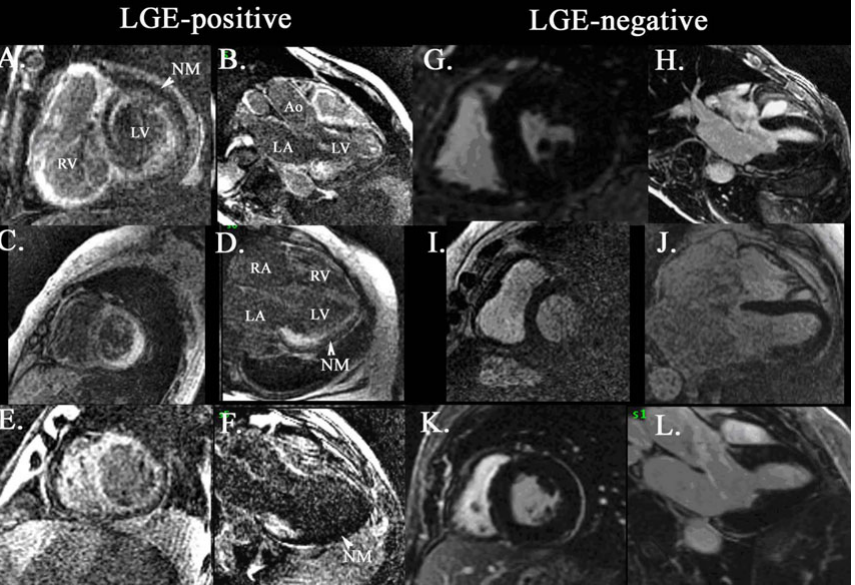
**Cardiac MRI**. Inversion-recovery prepared segmented gradient echo sequence short axis and long axis images of 3 patients with late gadolinium enhancement (A-B, C-D and E-F) and 3 patients without late gadolinium enhancement (G-H, I-J and K-L). Note the diffuse left ventricular subendocardial enhancement in LGE-positive patients and significant right ventricular involvement in the first two subjects. The dark nulled normal myocardium is located in the subepicardial portion. The dark blood pool signal in LGE-positive subjects is consistent with initial observations by Maceira, et al. [6] that they related to high myocardial uptake and fast blood washout. LV-left ventricle, RV-right ventricle, LA-left atrium, RA-right atrium, Ao-aorta, NM-nulled myocardium.

Late gadolinium enhancement was present in 23 (79%) of patients (Figure 2). The pattern was subendocardial in all 23 patients with varying degrees of transmurality. The distribution was found to be global in most cases (Figure 2A–D) but in some cases was confined to the basal inferolateral (Figure 2E–F). Left ventricular ejection fraction was similar between LGE-positive and LGE-negative groups (66 ± 17 vs. 69 ± 12%, respectively, p = 0.8) (Table 1). The inferolateral walls were thicker and left ventricular mass index were higher in LGE-positive patients.

### Clinical Outcomes

**Figure 3:**
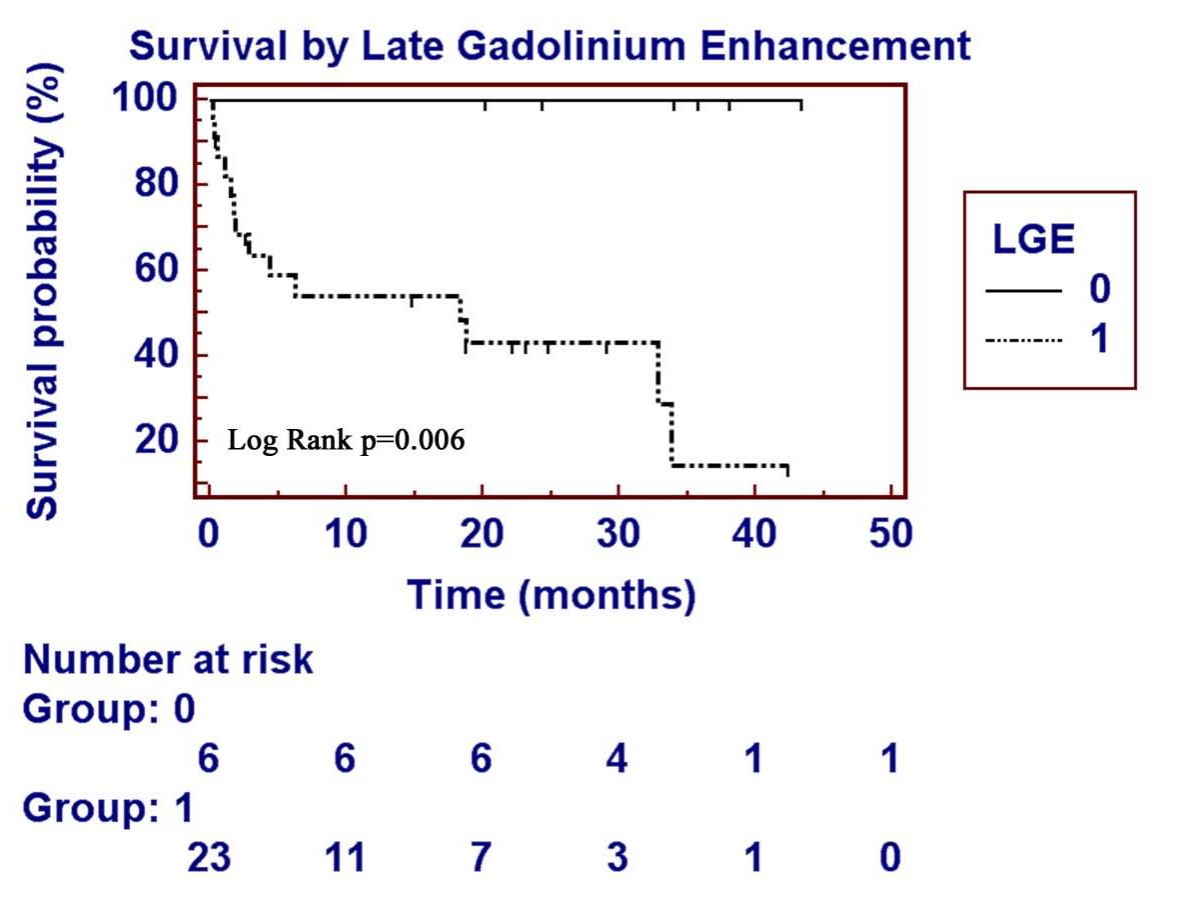
**Late gadolinium enhancement and survival**. Survival plot of light chain amyloid patients with (group 1) and without (group 0) late gadolinium enhancement showing significantly reduced survival in LGE-positive patients.

**Figure 4:**
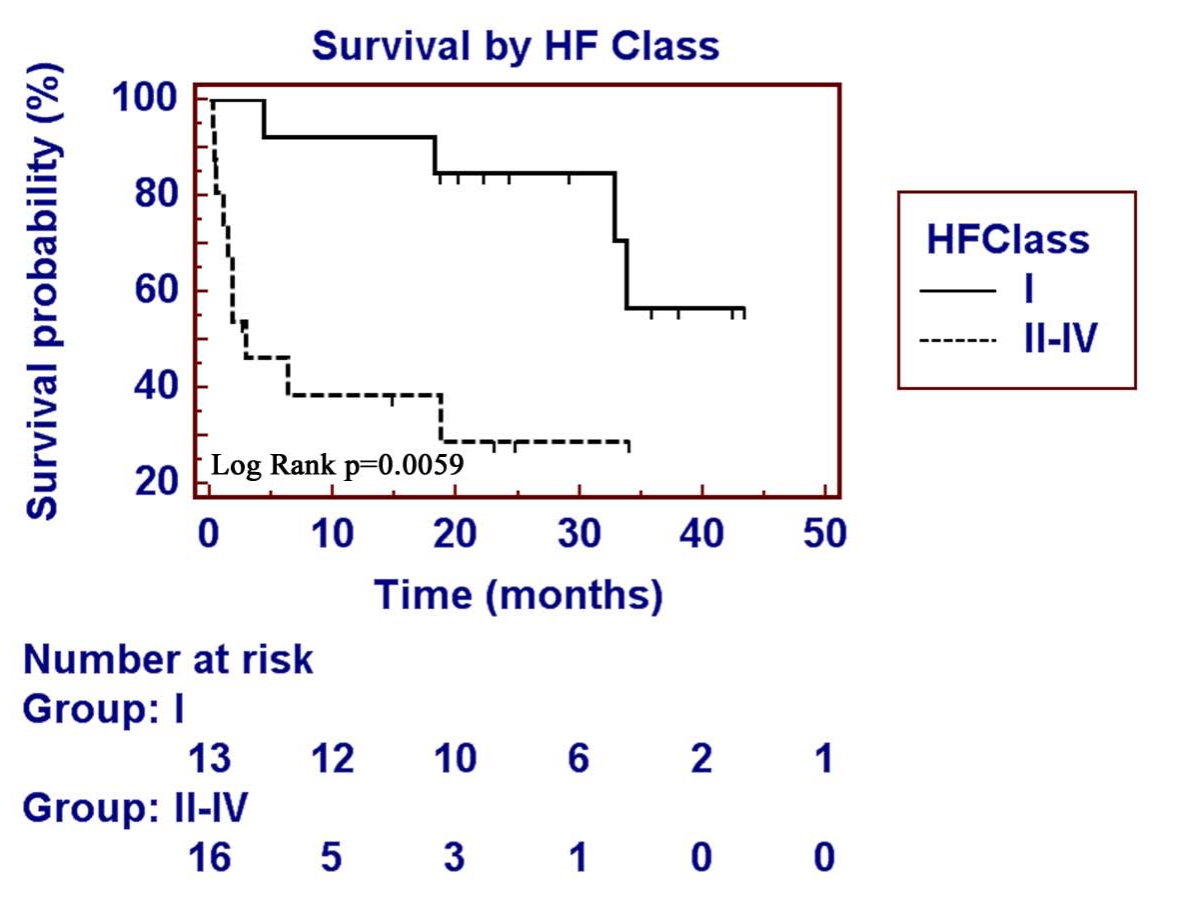
**Heart failure class and survival**. Survival plot of light chain amyloid patients with NYHA functional class I (no heart failure) and class II–IV (with heart failure). There is improved survival in patients in class I.

Follow-up of survival status was done 26 ± 13 (median 29) months following cardiac MRI. Overall 1-year mortality was 36% (46% for LGE-positive patients). The immediate cause of mortality was cardiac-related in the majority of patients (Table 2). Among variables tested by univariate log-rank analysis, heart failure class and late gadolinium enhancement were the strongest predictors of mortality (Table 3). Other variables that were significant included low voltage on electrocardiogram and inferolateral thickness. Left ventricular ejection fraction was not predictive of outcome. During the follow up period, 14 of 23 LGE-positive and 0 of 6 LGE-negative subjects died (log rank p = 0.0061, Figure 3). Presenting New York Heart Association class was a significant univariate predictor of outcomes (overall log rank p = 0.0059) and patients with Class I heart failure fared better than Class II–IV (Figure 4). Among those without presenting heart failure (Class I), 4 of 8 LGE-positive and 0 of 5 LGE-negative patients died during follow up. Exact log-rank test comparing two LGE groups stratified by heart failure class still showed a significant difference (p = 0.04).

**Table 2:** Causes of mortality

Subject	Cause	Interval from MRI (months)
1	Multiorgan failure	0.4

2	Renal failure	1.8

3	Sudden cardiac death	2.9

4	Pulmonary and heart failure	18.8

5	Multiorgan failure	1.5

6	Electromechanical dissociation, heart failure	0.5

7	Refractory multiple myeloma	18.3

8	Multiorgan failure	32.8

9	Unknown*	4.4

10	Heart failure	6.3

11	Sudden cardiac death	1.1

12	Pulseless electrical activity	34

13	Heart failure, ventricular tachycardia, pneumonia	1.8

14	Ventricular tachycardia, complete heart block	0.23

*Death was verified from Social Security Death index. Exact cause of death could not be verified.

**Table 3:** Univariate predictors of mortality.

Variable	χ^2^	p-value
NYHA Heart Failure Class	13.3	0.004

Late gadolinium enhancement	7.5	0.006

Inferolateral thickness > 1.2 cm	5.7	0.02

ECG low voltage	4.4	0.03

Alkaline phosphatase > 104 U/L	3.7	0.06

Left ventricular mass index > 70 g/m^2^	3.3	0.07

Troponin > 0.1 μg/mL	2.8	0.09

Anteroseptal thickness > 1.2 cm	1.2	0.26

Left ventricular ejection fraction < 60%	0.008	0.93

## Discussion

The study's novel observation is that the presence of late gadolinium enhancement on cardiac MRI is associated with poor survival on long-term follow up in light chain amyloidosis patients and that this MRI finding had independent and additive value to clinical assessment of heart failure status. This may be useful in identifying high risk patients and adds prognostic value to the known diagnostic value of LGE in light chain amyloidosis.

### LGE in AL

Light chain amyloidosis is a rare condition but cardiac involvement is important. Median survival following standard chemotherapy was reported to be 12–18 months but with heart failure, the survival is reduced to 4 months [4,5]. Light chain amyloid heart disease is associated with significant diastolic dysfunction that results from amyloid deposition [13,14]. Recently, it was reported that patients with cardiac amyloidosis demonstrate LGE on cardiac MRI that correlates closely with biopsy evidence of amyloid deposition in the myocardium [6,7]. Although light chain amyloidosis is a multi-organ disease, prior reports show that the heart is involved in ~50% of cases [3]. Based on LGE, our cohort of subjects had a higher proportion of cardiac involvement, although we lack myocardial biopsy confirmation in a majority of our subjects (local practice by our oncologists preclude myocardial biopsy if biopsy in more accessible organs already establish the diagnosis in order to reduce patient risk, an approach similar to that of major amyloid centers [8,15]). The proportion with LGE is similar to the frequency reported by Maceira and colleagues (69%) [6]. Our results similarly demonstrate predominant diffuse subendocardial distribution of LGE, although some cases were confined to the basal inferolateral region.

Late gadolinium enhancement and presenting heart failure class were the strongest univariate predictors of mortality in our cohort. Heart failure is known to be associated with poor outcomes in these patients [4,5]. Stratified by heart failure class, LGE remains predictive of mortality, suggesting additional prognostic value to clinical presentation. Other univariate predictors of mortality include low voltage on electrocardiogram and inferolateral thickness, markers indicative of degree of myocardial involvement. Light chain amyloidosis is a rare disease and our single-center study is limited by the small sample size. Despite the high proportion of mortality in this cohort, the small sample size and absolute number of mortality preclude Cox multivariable modeling to assess the independent interactions among the variables, a significant limitation of the study. The small sample size leads to unstable parameter estimates and the absence of mortality in LGE-negative subjects produces non-estimable hazard ratios in Cox multivariable techniques. Therefore the results of the study need to be validated in multicenter studies involving larger number of patients. Our work represents a long-term study of the outcomes of light chain amyloidosis patients following cardiac MRI. The current study adds strength to the value of LGE in the work up of light chain amyloidosis: it is useful not only as a diagnostic tool with high sensitivity and specificity for detecting cardiac involvement as reported by Vogelsberg and colleagues [7] but in addition, the presence of LGE has adverse long-term prognostic implication that may be useful for risk-stratification. In contrast, Maceira and colleagues [8] recently reported that the presence of LGE in AL subjects had a trend, but not statistically significant, of reduced survival compared to those without LGE. However, when they used the T1 difference between subepicardium and subendocardium 2 minutes post-gadolinium injection, they noted worse prognosis if the difference was lower than 23 ms. They attributed the difference in prognostic value of gross presence of LGE versus quantification of gadolinium kinetics in AL by the superior discrimination by gadolinium kinetics for the severity and transmurality of amyloid burden. Our protocol did not allow us to measure the intramyocardial T1 difference in our cohort so this study is not able to confirm their findings.

### Potential Mechanism of Adverse Outcomes in AL

The mechanism underlying adverse outcome in patients with late gadolinium enhancement, a marker of cardiac amyloid involvement, is not established but may lie in tissue toxicity from light chain amyloid deposition. Autopsy studies reveal that the presence of amyloid in the perivascular and interstitial space is associated with histologic evidence of myocardial ischemia in 74% of patients [16]. Epicardial coronary perivascular infiltration is seen in 97% of coronaries that are predominantly but not exclusively non-obstructive [17]; patients present with angina, have impaired myocardial flow reserve and elevated troponin, indicating myocyte necrosis [4,18-21]. In colon specimens, markers of oxidative tissue stress such as lipid peroxidation products (hydroxynonenal and thiobarbituric acid reactive substances) and protein carbonyls were present surrounding amyloid deposits [22]. In rodent models, brief exposure to light chains derived from light chain amyloid patients caused oxidative stress leading to cardiomyocyte dysfunction [23,24]. Amyloid deposition in the heart is associated with diastolic dysfunction [10,13,14] but recently, systolic dysfunction in the form of intraventricular dyssynchrony has been increasingly recognized [15,25]. Recent data also suggest endothelial dysfunction in peripheral and coronary arterioles exposed to AL light chains associated with oxidative stress that may be contributing to microvascular disease [26,27].

## Conclusion

In conclusion, late gadolinium enhancement on cardiac magnetic resonance imaging is associated with poor long-term survival in light chain AL amyloidosis, even adjusted for heart failure class presentation. This finding may be useful in identifying high risk patients and adds prognostic value to the known diagnostic value of LGE in light chain amyloidosis.

## Competing interests

The authors declare that they have no competing interests.

## Authors' contributions

RQM was involved in conception and design, subject enrollment, acquisition of data, analysis and interpretation of data, drafting and final approval of the manuscript. RC was involved in acquisition of data, interpretation of data, revision and final approval of the manuscript. AS was involved in statistical analysis and interpretation of data, revision and final approval of the manuscript. MB and ST were involved in subject enrollment, revision and final approval of the manuscript. PH was involved in conception and design, subject enrollment, acquisition and interpretation of data, revision and final approval of the manuscript.

## Pre-publication history

The pre-publication history for this paper can be accessed here:


